# Global trends in gastric cancer burden among adolescents and young adults: A population-based observational study using GBD 2021 data

**DOI:** 10.1097/MD.0000000000050506

**Published:** 2026-09-04

**Authors:** Maoqiang Liu, Zhen-Zhen Peng, Wei Liu, Tao Zhang

**Affiliations:** a Department of Oncology, People’s Hospital Affiliated to Chongqing Three Gorges Medical College, Chongqing, China; b Department of Gastrointestinal Surgery, The First Affiliated Hospital of Chongqing Medical University; Chongqing, China; c Department of Nursing, The First Affiliated Hospital of Chongqing Medical University, Chongqing, China; d Department of Oncology, Laboratory of Immunity, Inflammation & Cancer, The First Affiliated Hospital of Chongqing Medical University; Chongqing, China; e Chongqing Key Laboratory of Development and Utilization of Genuine Medicinal Materials in Three Gorges Reservoir Area, Chongqing Three Gorges Medical College, Chongqing, China.

**Keywords:** adolescents and young adults, age–period–cohort model, gastric cancer, Global Burden of Disease, Socio-demographic Index

## Abstract

Gastric cancer (GC) in adolescents and young adults (AYA; 15–39 years) is rare but clinically distinct. Contemporary global estimates and temporal drivers – particularly age–period–cohort (APC) effects and socioeconomic differentials – remain unclear. We conducted a secondary analysis using data from the Global Burden of Disease 2021 database and quantified the incidence, mortality, and disability-adjusted life years of AYA GC across 204 countries and territories from 1990 to 2021. Age-standardized rates and estimated annual percentage changes (EAPCs) were calculated using log-linear regression. APC models were used to describe age-, period-, and cohort-related patterns. Disease burden was stratified by Socio-demographic Index (SDI), and Global Burden of Disease (GBD)-attributed deaths related to diets high in sodium and smoking were examined within the GBD comparative risk assessment framework. From 1990 to 2021, the global AYA GC burden declined substantially, yet disparities by sex, SDI, and cohort persisted. Globally, the age-standardized incidence rate of AYA GC decreased from 2.45 per 1,00,000 population in 1990 to 1.41 per 1,00,000 population in 2021, with an EAPC of −2.04% (95% confidence interval [CI]: −2.21 to −1.88). Deaths declined from 38.15 × 10^3^ to 24.27 × 10^3^, with an EAPC of −2.75% (95% CI: −2.94 to −2.57). The age-standardized disability-adjusted life year rate decreased from 99.73 to 46.64 per 1,00,000 population, with an EAPC of −2.54% (95% CI: −2.63 to −2.46). Males consistently had higher burdens than females. Incidence was positively correlated with SDI over time (*r* = 0.2393, *P* < .001), whereas mortality was negatively correlated with SDI in the 2021 cross-sectional analysis (*r* = −0.3092, *P* < .001). APC analysis showed a positive age effect peaking at 35 to 39 years and a net drift of −2.18% per year. Within the GBD comparative risk assessment framework, the GBD-attributed proportion of AYA GC deaths related to diets high in sodium was approximately 15% in East Asia in 2021, while the GBD-attributed proportion related to smoking exceeded 10% in parts of Central and Eastern Europe.

**Key Points**

• Global incidence, mortality, and DALY rates for AYA gastric cancer fell markedly from 1990 to 2021.
• APC modeling revealed a persistent positive age effect at 35–39 years and an overall net drift of −2.18% per year.
• Incidence remained higher – but declined faster – in high-SDI regions; mortality correlated negatively with SDI cross-sectionally.

## 1. Introduction

Gastric cancer (GC) remains a significant component of the global cancer burden.^[1]^ Although incidence and mortality have declined in recent decades in many settings,^[2]^ GC continues to be among the leading causes of cancer-related deaths worldwide, with particularly high rates in East Asia (e.g., China, South Korea, and Japan).^[2,3]^ Geographic variation reflects multiple factors, including dietary patterns, *Helicobacter pylori* infection, and environmental and host-related influences.^[3,4]^ Despite progress in screening and early detection in some high-income settings, the burden remains substantial in many low- and middle-income regions, underscoring persistent global inequities in prevention and control.^[5]^

Most epidemiologic research and control strategies for GC have focused on older populations, where absolute incidence is highest and screening programs are more established. In contrast, GC among adolescents and young adults (AYA; 15–39 years) has received comparatively less attention in population-level assessments.^[6]^ This gap is important because AYA GC has been reported to present with distinct clinicopathological features and poorer outcomes in some studies.^[7,8]^ Even when incidence is relatively low, disease occurring in early adulthood can carry a disproportionate individual and societal impact due to potential years of life lost and long-term disability.^[9]^ A clearer understanding of temporal patterns and global heterogeneity in AYA GC burden is therefore needed.

Describing overall trends alone, however, may not capture the underlying time-related dynamics or the socioeconomic context in which burden changes occur. Age–period–cohort (APC) models provide a structured approach to disentangle age-related patterns from period- and cohort-related changes, offering additional insight into temporal dynamics beyond simple time-trend analyses. In parallel, stratification by the Socio-demographic Index (SDI) enables characterization of ecological socioeconomic gradients and highlights disparities across development levels, which may inform priority-setting and hypothesis generation.

Accordingly, using Global Burden of Disease (GBD) 2021 estimates, we quantified the global, regional, and national burden of GC among AYA (15–39 years) from 1990 to 2021, including age-standardized incidence, mortality, and DALYs. We assessed temporal trends using estimated annual percentage change (EAPC), examined socioeconomic heterogeneity through SDI stratification, and applied APC modeling to describe age-, period-, and cohort-related patterns.

## 2. Methods

### 2.1. Study population and data collection

This was a secondary analysis of population-level estimates based on the Global Burden of Disease Study (GBD) 2021, developed by the Institute for Health Metrics and Evaluation (IHME), covering the period from 1990 to 2021. The GBD 2021 database provides comprehensive, comparable estimates of disease burden for 369 diseases and injuries across 204 countries and territories from 1990 to 2021.^[10]^ Specifically, we focused on individuals aged 15 to 39 years, defined as AYAs, to assess the global, regional, and national burden of GC in this population.

The primary indicators analyzed included incidence, mortality, and disability-adjusted life years (DALYs).^[11]^ All data were retrieved through the Global Health Data Exchange (GHDx) query tool (http://ghdx.healthdata.org/gbd-results-tool), a publicly accessible platform that hosts GBD data and results. All estimates were reported with 95% uncertainty intervals (UIs), calculated as the 2.5th and 97.5th percentiles of 1000 draws from the posterior distribution of each modeled estimate. These UIs account for uncertainties arising from data sparsity, modeling assumptions, and input variability.^[10]^

### 2.2. Study metrics

This study evaluated the AYA GC burden and trends using various metrics.^[11]^ To ensure comparability across regions and time, all metrics were standardized to account for age distribution differences. ASIR and age-standardized mortality rate denote age-standardized incidence and mortality rates, respectively, and were calculated using the GBD world standard population. DALYs (disability-adjusted life years) were calculated as the sum of years of life lost due to premature mortality and years lived with disability. EAPC (estimated annual percentage change) summarizes the average annual change in an age-standardized rate over a specified period based on log-linear regression. Uncertainty interval refers to the 95% uncertainty interval reported by GBD (2.5th–97.5th percentiles across 1000 draws).^[12]^

### 2.3. Estimated annual percentage change

To quantify the temporal trends in the burden of GC among AYAs from 1990 to 2021, the EAPC was calculated for age-standardized rates (ASRs) of incidence, mortality, and DALYs. The EAPC is a widely used summary measure that reflects the average annual rate of change in ASRs over a specified period, offering a standardized approach for trend analysis across countries and regions. A log-linear regression model was applied to estimate the EAPC. Specifically, the natural logarithm of the ASR was modeled as a linear function of calendar year (*x*): ln(ASR) = α + β*x* + ε, where β represents the annual change in the logarithmic ASR. The EAPC was then calculated as: EAPC = 100 × (exp(β) − 1).^[13,14]^

The corresponding 95% confidence intervals (CIs) for the EAPC were also computed. Interpretation of the trends was based on the EAPC and its 95% CI: If both the EAPC and the upper bound of the CI were <0, the ASR was considered to be decreasing. If both the EAPC and the lower bound of the CI were >0, the ASR was considered to be increasing. If the 95% CI included 0, the ASR was considered to be stable over time. All estimates were reported with 95% uncertainty intervals (UIs), derived from the 2.5th and 97.5th percentiles of 1000 draws from the posterior distribution of the model, ensuring robustness and reproducibility of the trend analysis.^[15]^

### 2.4. Socio-demographic Index

The SDI, developed by the IHME, is a composite measure reflecting income per capita, average educational attainment (ages ≥ 15), and total fertility rate (under age 25). Based on SDI values, countries are classified into 5 levels: low, low-middle, middle, high-middle, and high.^[16]^

In this study, SDI was used to stratify regions and explore disparities in the burden of GC among AYAs (aged 15–39). SDI classifications were obtained from the IHME database (https://ghdx.healthdata.org/search/site/SDI). We further assessed associations between SDI and incidence, mortality, and DALY rates using linear regression, Pearson correlation, and nonlinear modeling to better understand the impact of socioeconomic development on disease burden.^[17]^

### 2.5. Age–period–cohort (APC) models

The APC model was used to evaluate age, period, and birth-cohort patterns in AYA GC burden.^[18]^ APC analyses were performed using incident case counts and corresponding population denominators. Data preparation and post-processing were conducted using R software (version 4.2.3; R Foundation for Statistical Computing), and APC modeling was performed using the NIH/NCI Age–Period–Cohort Web Tool.

The overall descriptive and EAPC analyses in this study used annual GBD 2021 estimates from 1990 to 2021. In contrast, the APC analysis required equal-width age and period intervals. Therefore, individuals aged 15 to 39 years were grouped into 5 consecutive 5-year age bands: 15 to 19, 20 to 24, 25 to 29, 30 to 34, and 35 to 39 years. Calendar years were aggregated into 6 complete 5-year periods: 1992 to 1996, 1997 to 2001, 2002 to 2006, 2007 to 2011, 2012 to 2016, and 2017 to 2021. The incomplete 1990 to 1991 interval was not included in the APC model because it did not constitute a complete 5-year period.^[19]^

Birth cohorts were derived from calendar period and attained age and were labeled according to the midpoint values generated by the APC tool. Because of the inherent non-identifiability of age, period, and cohort effects, we reported the standard estimable APC functions generated by the tool, including net drift, local drift, the longitudinal age curve, and period and cohort relative risks. Net drift represents the overall log-linear annual percentage change in the expected age-adjusted rates, whereas local drift represents age-specific annual percentage changes. Period effects reflect time-related influences affecting all age groups, while cohort effects reflect differences across birth cohorts. Period and cohort relative risks were estimated relative to the central reference categories specified by the APC tool. Statistical significance was assessed using Wald tests, and APC estimates were reported with 95% confidence intervals.^[20]^

### 2.6. Risk factor attribution

We extracted GBD-attributed GC deaths and population attributable fractions among AYAs aged 15 to 39 years from the GBD 2021 Comparative Risk Assessment framework, focusing on 2 selected risk factors: diet high in sodium and smoking. Location-, year-, sex-, and age-specific estimates with 95% uncertainty intervals were obtained from the GBD 2021 database.

In this study, population attributable fractions were used to describe the proportions of AYA GC deaths attributed to these risk factors within the GBD comparative risk assessment framework. These estimates should be interpreted as GBD-modeled attributable burdens rather than causal fractions newly estimated from individual-level data in the present analysis. We compared the levels and temporal trends of these GBD-attributed proportions across regions and SDI categories to describe population-level patterns.^[10,21]^

### 2.7. Statistical analysis

Statistical analyses were performed using R software version 4.2.3. Two-sided tests were used, with statistical significance set at *P* < .05. GBD-modeled estimates, including incidence, deaths, mortality rates, DALYs, DALY rates, and GBD-attributed risk factor estimates, were reported with 95% uncertainty intervals (UIs), defined as the 2.5th and 97.5th percentiles of 1000 posterior draws. EAPC estimates, Pearson correlation coefficients, and APC model outputs were reported with 95% CIs. EAPC was used to summarize the average annual change in ASRS. Pearson correlation and linear regression analyses were used to evaluate ecological associations between SDI and AYA GC burden. Because this study was based on aggregate, population-level GBD estimates, the results were interpreted as descriptive and ecological patterns rather than individual-level causal associations.^[22]^

## 3. Results

### 3.1. Global and regional trends in AYA gastric cancer

**Figure 1.**
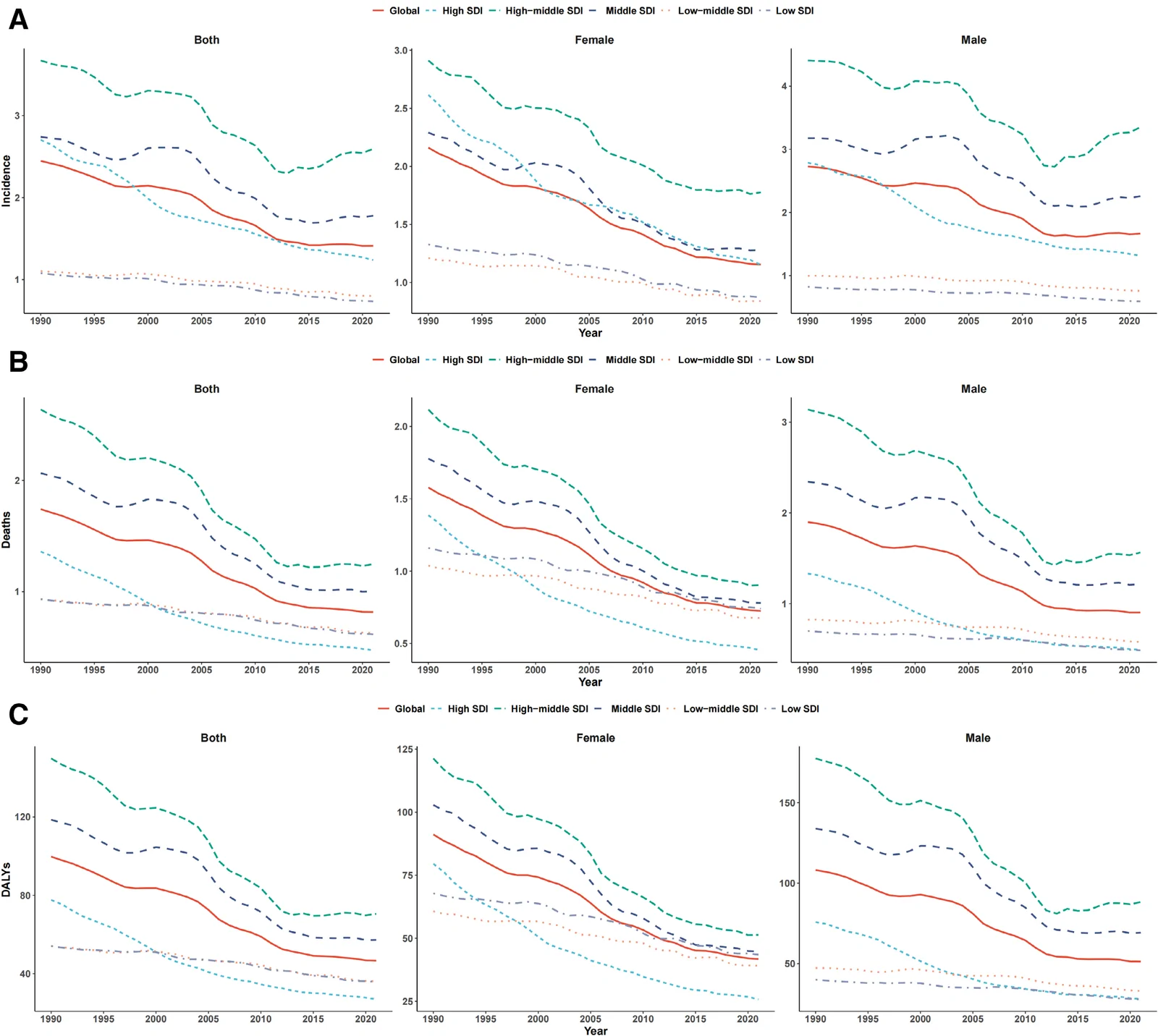
Temporal trends in incidence, mortality, and DALYs of AYA gastric cancer in SDI regions, 1990 to 2021. Legend: Panel (A) presents the global distribution of incidence rates across SDI quintiles (high, high-middle, middle, low-middle, and low SDI) and by sex (total, female, male). Panel (B) displays corresponding mortality rate trends, highlighting regional disparities. Panel (C) illustrates DALYs trends, reflecting the disease burden. All panels demonstrate declining global trends, with steeper reductions in high-SDI regions. Male AYAs consistently exhibited higher rates than females across all metrics. AYA = adolescents and young adults, DALY = disability-adjusted life year, SDI = Socio-demographic Index.

From 1990 to 2021, the global burden of AYA GC declined substantially. The global ASIR decreased from 2.45 (95% uncertainty interval: 2.13–2.67) per 1,00,000 in 1990 to 1.41 (1.21–1.59) per 1,00,000 in 2021 (EAPC: −2.04, 95% CI: −2.21 to −1.88; Table 1; Fig. 1A). Over the same period, the number of deaths declined from 38.15 (33.18–41.71) × 10^3^ to 24.27 (21.1–26.93) × 10^3^ (EAPC: −2.75, 95% CI: −2.94 to −2.57; Table 2; Fig. 1B). The age-standardized disability-adjusted life years rate decreased from 99.73 (86.82–108.85) per 1,00,000 in 1990 to 46.64 (40.45–51.71) per 1,00,000 in 2021 (EAPC: −2.54, 95% CI: −2.63 to −2.46; Table 3; Fig. 1C), indicating a marked global reduction in burden.

**Table 1:** Incidence rate and case numbers of AYA gastric cancer in 1990 and 2021 with EAPC from 1990 to 2021.

Measure	1990		2021		1990–2021
	Incidence per 1,00,000 (95% UI)	Number of incidence (95% UI, ×10^3^)	Incidence per 1,00,000 (95% UI)	Number of incidence (95% UI)	EAPC (95% CI)
location_name					
Global	2.45 (2.13, 2.67)	53638.74 (46760.81, 58447.38)	1.41 (1.21, 1.59)	42038.99 (35999.57, 47270.73)	−2.04 (−2.21, −1.88)
Sex					
Male	2.73 (2.13, 3.08)	30227.08 (23563.39, 34096.96)	1.66 (1.29, 1.95)	25124.97 (19400.88, 29460.28)	−1.61 (−1.69 to −1.53)
Female	2.16 (1.92, 2.44)	23411.66 (20766.20, 26399.25)	1.15 (1.02, 1.30)	16914.02 (14932.63, 19040.01)	−2.24 (−2.31 to −2.17)
SDI region					
High SDI	2.70 (2.46 ,2.81)	9379.32 (8529.42, 9765.43)	1.24 (1.18, 1.33)	4369.77 (4153.53, 4698.63)	−2.57 (−2.69, −2.44)
High-middle SDI	3.67 (3.15, 4.03)	16615.22 (14266.88, 18250.15)	2.59 (2.06, 3.02)	11421.50 (9090.62, 13301.73)	−1.55 (−1.78, −1.32)
Middle SDI	2.74 (2.34, 3.10)	20623.11 (17634.25, 23331.30)	1.78 (1.51, 2.07)	16496.58 (14027.63, 19222.38)	−1.79 (−2.04, −1.53)
Low-middle SDI	1.10 (0.93, 1.24)	5002.26 (4197.73, 5612.30)	0.80 (0.71, 0.91)	6421.16 (5663.83, 7309.00)	−1.09 (−1.19, −0.99)
Low SDI	1.08 (0.83, 1.26)	1988.88 (1522.66, 2330.12)	0.74 (0.58, 0.85)	3305.64 (2626.64, 3832.19)	−1.27 (−1.35, −1.18)
Geographic region					
Andean Latin America	3.03 (2.58, 3.46)	468.82 (398.84, 535.71)	2.44 (1.95, 2.99)	661.69 (526.98, 809.46)	−0.94 (−1.15, −0.72)
Australasia	1.03 (0.96, 1.10)	84.08 (78.15, 90.00)	0.73 (0.65, 0.80)	75.98 (67.84, 83.82)	−1.27 (−1.46, −1.09)
Caribbean	1.19 (1.02, 1.37)	177.29 (151.01, 203.16)	1.06 (0.84, 1.31)	193.83 (153.30, 237.94)	−0.32 (−0.49, −0.15)
Central Asia	3.01 (2.85, 3.17)	857.74 (809.94, 902.80)	1.46 (1.29, 1.68)	545.80 (481.95, 628.21)	−2.81 (−3.04, −2.58)
Central Europe	1.67 (1.60, 1.74)	782.82 (749.06, 816.39)	0.77 (0.70, 0.84)	268.02 (243.85, 292.50)	−2.38 (−2.55, −2.21)
Central Latin America	1.80 (1.73, 1.88)	1229.94 (1184.33, 1280.64)	1.77 (1.58, 1.97)	1788.60 (1594.33, 1992.08)	−0.20 (−0.30, −0.11)
Central Sub-Saharan Africa	0.86 (0.65, 1.09)	178.93 (135.75, 225.80)	0.63 (0.46, 0.83)	342.90 (249.90, 446.41)	−1.01 (−1.07, −0.95)
East Asia	4.65 (3.85, 5.39)	26308.33 (21773.75, 30517.73)	4.07 (3.18, 4.96)	19478.49 (15253.59, 23741.78)	−0.97 (−1.29, −0.64)
Eastern Europe	4.24 (4.12, 4.37)	3640.82 (3531.73, 3748.52)	2.09 (1.91, 2.26)	1383.44 (1265.35, 1496.63)	−2.55 (−2.83, −2.27)
Eastern Sub-Saharan Africa	1.02 (0.79, 1.23)	722.93 (560.93, 872.35)	0.62 (0.50, 0.75)	1087.80 (883.04, 1316.81)	−1.84 (−1.96, −1.72)
High-income Asia Pacific	8.36 (7.26, 8.87)	5641.83 (4901.01, 5984.01)	3.05 (2.77, 3.49)	1540.41 (1399.85, 1765.93)	−3.14 (−3.33, −2.96)
High-income North America	0.95 (0.93, 0.97)	1076.06 (1051.43, 1099.03)	0.90 (0.87, 0.93)	1105.75 (1065.85, 1147.46)	−0.31 (−0.45, −0.17)
North Africa and Middle East	1.42 (1.07, 1.64)	1901.13 (1436.41, 2194.26)	0.99 (0.68, 1.17)	2511.56 (1719.65, 2972.85)	−1.03 (−1.15, −0.92)
Oceania	2.53 (1.52, 3.33)	67.33 (40.34, 88.49)	2.36 (1.74, 3.09)	132.80 (97.84, 174.25)	−0.37 (−0.46, −0.28)
South Asia	0.88 (0.73, 1.03)	3818.97 (3163.23, 4441.83)	0.60 (0.52, 0.70)	4709.21 (4148.84, 5567.18)	−1.27 (−1.42, −1.11)
Southeast Asia	1.33 (1.04, 1.54)	2616.03 (2047.12, 3025.91)	0.96 (0.79, 1.14)	2673.29 (2203.22, 3155.74)	−1.40 (−1.61, −1.19)
Southern Latin America	1.55 (1.45, 1.64)	295.50 (277.45, 313.69)	1.06 (0.97, 1.17)	274.50 (249.97, 301.82)	−1.05 (−1.19, −0.91)
Southern Sub-Saharan Africa	1.11 (0.96, 1.26)	240.21 (207.98, 271.30)	0.80 (0.66, 0.98)	273.27 (225.20, 334.44)	−1.03 (−1.69, −0.36)
Tropical Latin America	1.52 (1.46, 1.58)	979.21 (942.00, 1018.55)	1.26 (1.21, 1.32)	1114.39 (1064.62, 1166.39)	−0.81 (−0.92, −0.70)
Western Europe	1.47 (1.43, 1.53)	2124.28 (2056.55, 2199.98)	0.76 (0.73, 0.80)	992.50 (948.49, 1037.65)	−2.24 (−2.39, −2.09)
Western Sub-Saharan Africa	0.60 (0.48, 0.70)	426.49 (341.42, 500.59)	0.46 (0.34, 0.56)	884.78 (657.24, 1073.17)	−0.64 (−0.73, −0.54)

This table displays the incidence rates (per 1,00,000 population) and total case numbers of AYA gastric cancer in 1990 and 2021, stratified by sex, SDI, and geographic regions. EAPC indicates the overall trend from 1990 to 2021. SDI levels are categorized as low, low-middle, middle, high-middle, and high, representing varying levels of social and economic development. All values are presented with 95% uncertainty intervals (UI), and EAPC is provided with 95% confidence intervals (CI).

AY = adolescents and young adults, CI = confidence interval, EAPC = estimated annual percentage change, SDI = Socio-demographic Index, UI = uncertainty interval.

**Table 2:** Mortality rate and case numbers of AYA gastric cancer in 1990 and 2021 with EAPC from 1990 to 2021.

Measure	1990		2021		1990–2021
	Mortality per 100 000 (95% UI)	Number of deaths (95% UI)	Mortality per 100 000 (95% UI)	Number of deaths (95% UI)	EAPC (95% CI)
location_name					
Global	1.74 (1.51, 1.90)	38152.16 (33182.79, 41714.95)	0.82 (0.71, 0.91)	24271.92 (21097.28, 26933.45)	−2.75 (−2.94, −2.57)
Sex					
Male	1.90 (1.46, 2.15)	21053.82 (16143.14, 23874.39)	0.90 (0.71, 1.05)	13639.37 (10697.44, 15894.28)	−2.80 (−3.03, −2.57)
Female	1.58 (1.39, 1.78)	17098.34 (15084.63, 19321.93)	0.73 (0.65, 0.81)	10632.55 (9491.82, 11821.97)	−2.70 (−2.82, −2.57)
SDI region					
High SDI	1.36 (1.22, 1.42)	4713.50 (4250.16, 4934.95)	0.47 (0.45, 0.51)	1668.75 (1586.93, 1803.88)	−3.55 (−3.74, −3.35)
High-middle SDI	2.64 (2.25, 2.91)	11932.21 (10199.16, 13150.14)	1.25 (1.02, 1.44)	5490.55 (4493.20, 6335.27)	−2.94 (−3.20, −2.67)
Middle SDI	2.06 (1.77, 2.33)	15541.28 (13324.08, 17501.54)	1.00 (0.88, 1.15)	9292.13 (8134.28, 10628.24)	−2.74 (−3.02, −2.47)
Low-middle SDI	0.93 (0.78, 1.04)	4220.57 (3542.98, 4726.68)	0.63 (0.55, 0.71)	5036.78 (4429.67, 5734.50)	−1.31 (−1.43, −1.20)
Low SDI	0.93 (0.71, 1.09)	1720.77 (1310.86, 2012.60)	0.62 (0.49, 0.72)	2765.41 (2204.25, 3222.43)	−1.38 (−1.48, −1.28)
Geographic region					
Andean Latin America	2.50 (2.12, 2.85)	386.16 (328.16, 441.13)	1.73 (1.38, 2.12)	468.81 (374.48, 573.74)	−1.43 (−1.65, −1.22)
Australasia	0.50 (0.47, 0.54)	41.16 (38.33, 43.98)	0.29 (0.26, 0.31)	30.01 (27.13, 32.76)	−1.97 (−2.17, −1.77)
Caribbean	0.95 (0.80, 1.09)	141.03 (118.44, 162.33)	0.84 (0.65, 1.03)	152.63 (118.37, 188.07)	−0.30 (−0.47, −0.13)
Central Asia	2.55 (2.40, 2.68)	724.75 (684.04, 763.94)	1.18 (1.04, 1.35)	440.74 (388.15, 505.89)	−2.98 (−3.20, −2.76)
Central Europe	1.34 (1.29, 1.40)	629.53 (602.71, 657.08)	0.54 (0.50, 0.59)	190.60 (174.22, 207.00)	−2.82 (−3.00, −2.65)
Central Latin America	1.44 (1.39, 1.49)	981.78 (945.91, 1020.06)	1.26 (1.12, 1.41)	1276.32 (1134.73, 1427.41)	−0.56 (−0.65, −0.46)
Central Sub-Saharan Africa	0.74 (0.57, 0.94)	154.39 (117.76, 194.65)	0.53 (0.39, 0.70)	286.68 (208.70, 375.99)	−1.10 (−1.15, −1.05)
East Asia	3.38 (2.81, 3.92)	19096.75 (15882.01, 22157.78)	1.85 (1.47, 2.26)	8875.78 (7025.74, 10817.95)	−2.59 (−2.98, −2.20)
Eastern Europe	2.99 (2.90, 3.08)	2562.07 (2483.59, 2638.74)	1.25 (1.13, 1.37)	826.50 (747.03, 905.12)	−3.18 (−3.40, −2.96)
Eastern Sub-Saharan Africa	0.88 (0.68, 1.06)	626.05 (483.40, 753.64)	0.52 (0.42, 0.63)	903.93 (732.11, 1099.03)	−1.97 (−2.09, −1.86)
High-income Asia Pacific	3.79 (3.17, 4.07)	2558.24 (2140.67, 2747.52)	0.90 (0.82, 1.02)	452.41 (413.40, 516.74)	−4.71 (−4.87, −4.55)
High-income North America	0.41 (0.40, 0.42)	461.82 (452.95, 470.56)	0.32 (0.31, 0.33)	394.55 (380.28, 408.80)	−0.93 (−1.09, −0.77)
North Africa and Middle East	1.21 (0.94,1.40)	1617.74 (1254.72, 1868.74)	0.76 (0.53, 0.92)	1928.13 (1348.65, 2334.63)	−1.37 (−1.47, −1.28)
Oceania	2.09 (1.27, 2.78)	55.59 (33.70, 73.80)	1.88 (1.39, 2.47)	106.00 (78.43, 139.40)	−0.44 (−0.53, −0.36)
South Asia	0.75 (0.62, 0.87)	3231.69 (2686.33, 3759.98)	0.47 (0.41, 0.55)	3713.14 (3271.20, 4389.17)	−1.49 (−1.66, −1.31)
Southeast Asia	1.09 (0.86, 1.26)	2138.02 (1687.28, 2480.42)	0.68 (0.56, 0.80)	1876.67 (1540.69, 2219.40)	−1.85 (−2.05, −1.65)
Southern Latin America	1.11 (1.04, 1.18)	212.33 (199.34, 226.02)	0.65 (0.59, 0.71)	167.67 (152.52, 183.01)	−1.53 (−1.68, −1.39)
Southern Sub-Saharan Africa	0.90 (0.78, 1.01)	193.78 (167.86, 218.98)	0.64 (0.53, 0.79)	217.83 (179.24, 267.27)	−1.04 (−1.71, −0.36)
Tropical Latin America	1.21 (1.16, 1.26)	777.83 (748.68, 807.34)	0.91 (0.87, 0.96)	806.82 (770.81, 843.52)	−1.10 (−1.20, −0.99)
Western Europe	0.83 (0.81, 0.86)	1197.63 (1162.61, 1237.11)	0.33 (0.31, 0.34)	424.85 (407.67, 443.49)	−3.13 (−3.24, −3.02)
Western Sub-Saharan Africa	0.51 (0.41, 0.60)	363.83 (292.74, 427.81)	0.38 (0.29, 0.46)	731.85 (546.57, 886.69)	−0.73 (−0.82, −0.64)

This table displays the mortality rates (per 1,00,000 population) and total case numbers of AYA gastric cancer in 1990 and 2021, stratified by sex, SDI, and geographic regions. EAPC indicates the overall trend from 1990 to 2021. SDI levels are categorized as low, low-middle, middle, high-middle, and high, representing varying levels of social and economic development. All values are presented with 95% uncertainty intervals (UI), and EAPC is provided with 95% confidence intervals (CI).

AYA = adolescents and young adults, CI = confidence interval, EAPC = estimated annual percentage change, SDI = Socio-demographic Index, UI = uncertainty interval.

**Table 3:** DALYs rate and case numbers of AYA gastric cancer in 1990 and 2021 with EAPC from 1990 and 2021.

Measure	1990		2021		1990–2021
	DALYs per 1,00,000 (95% UI)	Number of DALYs (95% UI)	DALYs per 1,00,000 (95% UI)	Number of DALYs (95% UI)	EAPC (95% CI)
location_name					
Global	99.73 (86.82, 108.85)	21,85,988.97 (19,02,869.69, 23,85,820.55)	46.64 (40.45, 51.71)	13,87,535.91 (12,03,370.15, 15,38,231.87)	−2.75 (−2.92, −2.57)
Sex					
Male	108.11 (82.87, 122.80)	11,98,374.73 (9,18,633.79, 13,61,187.31)	51.35 (40.28, 59.77)	7,75,250.69 (6,08,067.37, 9,02,282.46)	−2.79 (−3.01, −2.57)
Female	91.16 (80.45, 102.76)	9,87,614.24 (8,71,543.68, 11,13,265.70)	41.79 (37.23, 46.36)	6,12,285.22 (5,45,463.03, 6,79,193.80)	−2.70 (−2.82, −2.58)
SDI region					
High SDI	77.64 (69.86, 81.25)	2,69,374.08 (2,42,386.51, 2,81,897.35)	26.90 (25.59, 29.09)	95,029.75 (90,384.55, 1,02,770.93)	−3.54 (−3.74, −3.35)
High-middle SDI	149.86 (128.23, 165.08)	6,78,172.78 (5,80,285.28, 7,47,042.51)	70.57 (57.84, 81.26)	3,10,712.35 (2,54,669.08, 3,57,771.61)	−2.92 (−3.18, −2.67)
Middle SDI	118.61 (101.64, 133.51)	8,92,674.98 (7,65,009.41, 10,04,846.14)	57.21 (50.10, 65.28)	5,30,579.41 (4,64,651.90, 6,05,491.00)	−2.75 (−3.01, −2.50)
Low-middle SDI	53.96 (45.31, 60.45)	2,44,672.93 (2,05,414.51, 2,74,101.42)	36.07 (31.72, 41.00)	2,89,480.71 (2,54,563.43, 3,29,052.15)	−1.34 (−1.46, −1.23)
Low SDI	54.11 (41.23, 63.40)	99,725.63 (75,988.34, 1,16,842.84)	35.78 (28.60, 41.58)	1,60,678.41 (1,28,425.59, 1,86,707.38)	−1.39 (−1.49, −1.28)
Geographic region					
Andean Latin America	146.76 (124.31, 167.52)	22,694.88 (19,223.34, 25,904.49)	100.16 (80.14, 122.26)	27,122.57 (21,701.37, 33,107.51)	−1.47 (−1.69, −1.25)
Australasia	29.00 (27.06, 30.92)	2364.94 (2206.17, 2521.24)	16.32 (14.79, 17.79)	1708.67 (1548.64, 1863.24)	−1.99 (−2.19, −1.79)
Caribbean	55.20 (46.30, 63.51)	8204.79 (6882.77, 9441.17)	48.41 (37.63, 59.52)	8812.26 (6849.36, 10,833.59)	−0.31 (−0.48 , −0.14)
Central Asia	147.06 (138.78, 155.10)	41,843.01 (39,487.95, 44,131.07)	67.37 (59.26, 77.47)	25,187.97 (22,155.48, 28,963.71)	−3.00 (−3.21, −2.78)
Central Europe	75.63 (72.43, 78.86)	35,432.46 (33,931.54, 36,942.41)	30.52 (27.85, 33.11)	10,687.12 (9751.41, 11,594.16)	−2.83 (−3.00, −2.66)
Central Latin America	84.21 (81.07, 87.42)	57,490.02 (55,347.22, 59,682.22)	72.81 (64.80, 81.45)	73,653.28 (65,554.54, 82,397.99)	−0.60 (−0.69, −0.51)
Central Sub-Saharan Africa	43.19 (32.92, 54.40)	8967.81 (6834.54, 11,294.47)	30.74 (22.34, 40.15)	16,628.12 (12,086.86, 21,720.75)	−1.10 (−1.15, −1.05)
East Asia	192.44 (160.39, 223.23)	10,88,629.22 (9,07,323.66, 12,62,812.99)	105.21 (83.32, 127.89)	5,03,997.80 (3,99,124.18, 6,12,661.29)	−2.58 (−2.95, −2.21)
Eastern Europe	168.58 (163.48, 173.56)	1,44,592.01 (1,40,210.18, 1,48,862.98)	69.99 (63.28, 76.56)	46,312.94 (41,876.06, 50,660.22)	−3.17 (−3.38, −2.97)
Eastern Sub-Saharan Africa	51.41 (39.75, 61.86)	36,441.43 (28,179.24, 43,849.50)	30.01 (24.30, 36.46)	52,573.18 (42,574.67, 63,880.44)	−1.98 (−2.09, −1.87)
High-income Asia Pacific	217.60 (181.08, 233.34)	1,46,868.62 (1,22,217.74, 1,57,496.41)	51.33 (47.04, 58.57)	25,940.44 (23,773.60, 29,601.17)	−4.72 (−4.89, −4.56)
High-income North America	23.36 (22.92, 23.82)	26,472.57 (25,967.64, 26,991.27)	18.39 (17.73, 19.04)	22,659.03 (21,843.80, 23,459.85)	−0.90 (−1.05, −0.74)
North Africa and Middle East	70.50 (54.75, 81.39)	94,346.94 (73,268.10, 1,08,920.48)	43.79 (30.67, 52.98)	1,11,345.00 (77,976.96, 1,34,704.77)	−1.41 (−1.50, −1.32)
Oceania	122.70 (73.56, 162.09)	3259.40 (1953.96, 4305.71)	110.21 (81.45, 144.51)	6209.70 (4589.52, 8142.31)	−0.45 (−0.53, −0.36)
South Asia	43.14 (35.93, 50.08)	1,86,201.29 (1,55,090.90, 2,16,151.22)	26.85 (23.66, 31.70)	2,12,326.16 (1,87,161.35, 2,50,727.70)	−1.52 (−1.70, −1.35)
Southeast Asia	63.34 (49.90, 73.31)	1,24,783.09 (98,307.57, 1,44,429.90)	38.93 (32.01,45.96)	1,07,949.87 (88,759.06, 1,27,459.05)	−1.90 (−2.10, −1.70)
Southern Latin America	63.79 (60.01, 67.68)	12,171.18 (11,450.09, 12,913.40)	37.33 (33.96, 40.75)	9629.34 (8759.92, 10,512.94)	−1.53 (−1.67, −1.39)
Southern Sub-Saharan Africa	51.46 (44.60, 58.08)	11,123.27 (9641.29, 12,555.08)	36.47 (30.07, 44.55)	12,414.20 (10,236.07, 15,162.86)	−1.05 (−1.73, −0.36)
Tropical Latin America	69.56 (66.89, 72.13)	44,733.68 (43,021.94, 46,386.57)	52.06 (49.75, 54.47)	45,977.98 (43,931.03, 48,106.17)	−1.12 (−1.22, −1.02)
Western Europe	47.38 (46.06, 48.92)	68,290.65 (66,379.75, 70,498.30)	18.50 (17.73, 19.29)	24,006.24 (23,010.32, 25,028.74)	−3.16 (−3.26, −3.06)
Western Sub-Saharan Africa	29.45 (23.69, 34.65)	21,077.72 (16,953.58, 24,798.78)	22.17 (16.59, 26.85)	42,394.03 (31,730.54, 51,345.38)	−0.73 (−0.81, −0.64)s

This table displays the DALYs rates (per 1,00,000 population) and total case numbers of AYA gastric cancer in 1990 and 2021, stratified by sex, SDI, and geographic regions. EAPC indicates the overall trend from 1990 to 2021. SDI levels are categorized as low, low-middle, middle, high-middle, and high, representing varying levels of social and economic development. All values are presented with 95% uncertainty intervals (UI), and EAPC is provided with 95% confidence intervals (CI).

AYA = adolescents and young adults, CI = confidence interval, DALY = disability-adjusted life years, EAPC = estimated annual percentage change, SDI = Socio-demographic Index, UI = uncertainty interval.

Gender-stratified analyses showed consistently higher burdens among males than females across outcomes (Tables 1–3; Fig. 1A–C). Incidence decreased from 2.73 (2.13–3.08) to 1.66 (1.29–1.95) per 1,00,000 in males (EAPC −1.61, 95% CI: −1.69 to −1.53) and from 2.16 (1.92–2.44) to 1.15 (1.02–1.30) per 1,00,000 in females (EAPC: −2.24, 95% CI: −2.31 to −2.17; Table 1; Fig. 1A). Deaths declined from 21.05 (16.14–23.87) × 10^3^ to 13.64 (10.7–15.89) × 10^3^ in males (EAPC: −2.80, 95% CI: −3.03 to −2.57) and from 17.1 (15.08–19.32) × 10^3^ to 10.63 (9.49–11.82) × 10^3^ in females (EAPC: −2.70, 95% CI: −2.82 to −2.57; Table 2; Fig. 1B). DALY rates declined from 108.11 (82.87–122.80) to 51.35 (40.28–59.77) per 1,00,000 in males (EAPC: −2.79, 95% CI: −3.01 to −2.57) and from 91.16 (80.45–102.76) to 41.79 (37.23–46.36) per 1,00,000 in females (EAPC: −2.70, 95% CI: −2.82 to −2.58; Table 3; Fig. 1C).

### 3.2. AYA gastric cancer burden across 204 countries and regions

**Figure 2.**
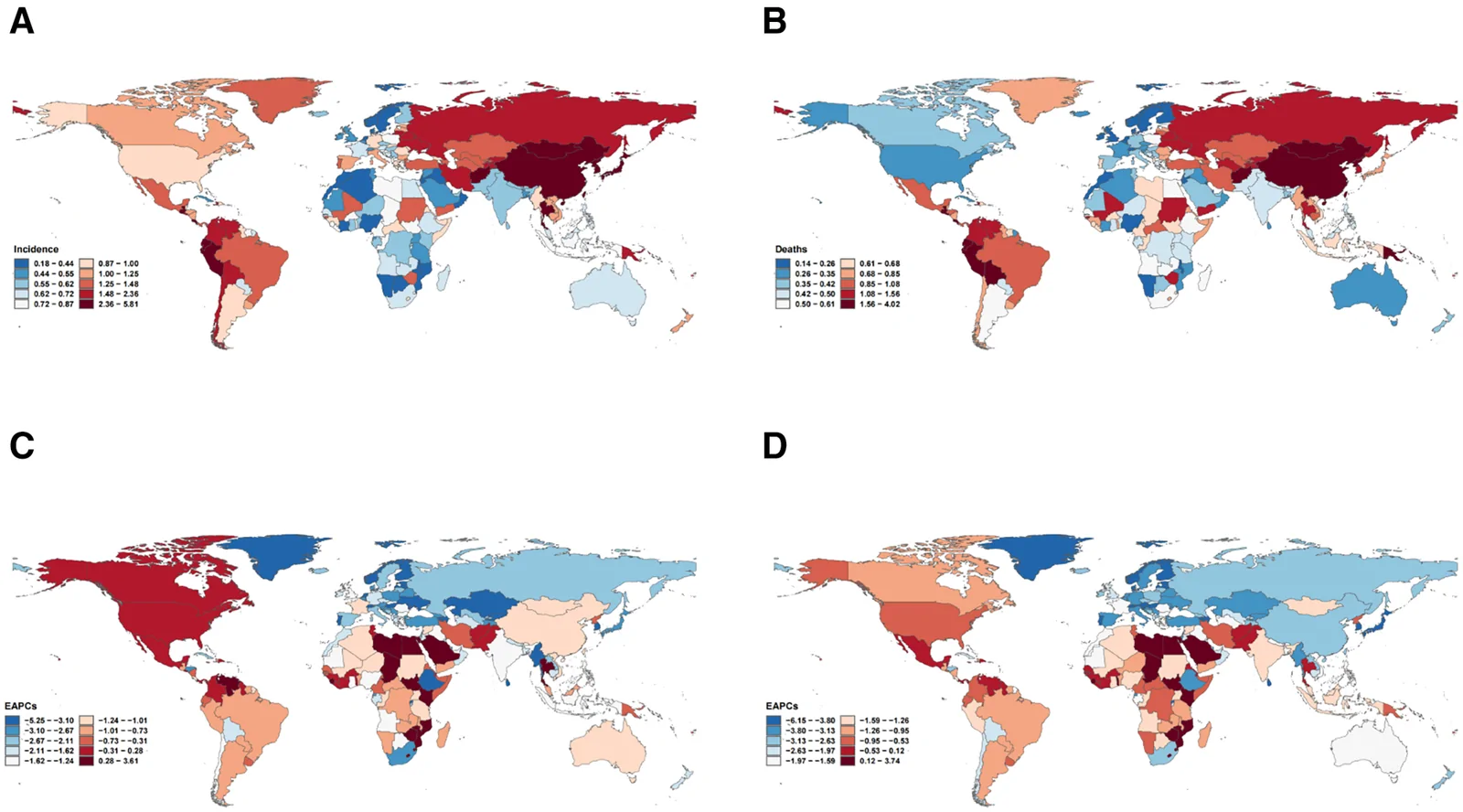
Incidence, mortality rate, and EAPC of AYA Gastric Cancer in 204 countries and territories. This figure illustrates the incidence rate, mortality rate, and estimated annual percentage change (EAPC) of AYA gastric cancer across 204 countries and territories. Panel (A) displays the global distribution of AYA gastric cancer incidence rates in 2021. Panel (B) presents the global distribution of AYA gastric cancer mortality rates in 2021. Panels (C) and (D) show the EAPC values for incidence and mortality rates from 1990 to 2021, respectively. Regions with positive or minimal EAPC values indicate increasing or stable trends, whereas negative EAPC values indicate declining rates over time. AYA = adolescents and young adults, EAPC = estimated annual percentage change.

In 2021, marked geographic heterogeneity persisted. Higher incidence and mortality were concentrated in several high-burden clusters, including parts of East Asia and Central Asia, whereas many high-income regions in Western/Northern Europe showed comparatively lower rates (Fig. 2A, B). Country-level extremes illustrate this heterogeneity: Palau had the highest incidence rate (5.75 [4.38–7.52] per 1,00,000), and Palau and Kiribati were among the highest in mortality (Fig. 2A, B).

From 1990 to 2021, most countries experienced declining incidence and mortality, with especially pronounced reductions across many parts of Europe, Southeast Asia, and South America (Fig. 2C, D). The largest decreases were observed in a small number of countries (e.g., Luxembourg and Saint Kitts and Nevis for incidence; Republic of Korea and Luxembourg for mortality), whereas some countries in Africa showed limited improvement or increases in mortality over time (Fig. 2D). Overall, these results highlight substantial cross-country variation in baseline burden and in the pace of change, reinforcing the importance of interpreting trends within a regional context (Fig. 2).

### 3.3. Regional and gender differences in AYA gastric cancer burden

**Figure 3.**
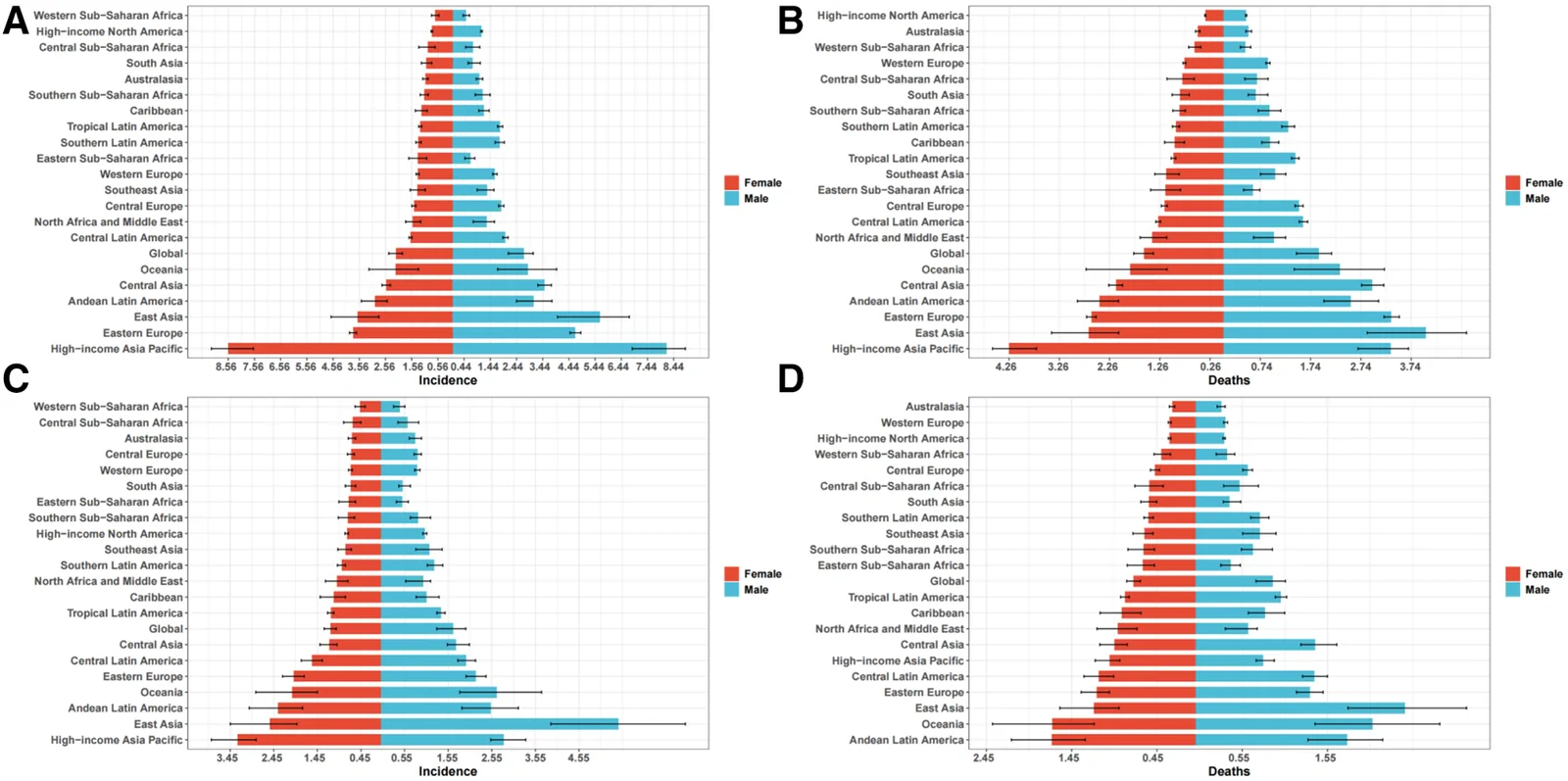
Regional incidence and mortality rates of AYA gastric cancer by sex in 21 GBD regions, 1990 and 2021. This figure illustrates the regional variation in the burden of gastric cancer among adolescents and young adults (AYAs) aged 15 to 39 across 21 Global Burden of Disease (GBD) regions, stratified by sex (female in red; male in blue) and year (Panels A and B for 1990, Panels C and D for 2021). Panels A and B represent the incidence and mortality rates of AYA gastric cancer in 1990, respectively, while panels C and D display these metrics for 2021. Error bars represent 95% uncertainty intervals (UIs). Across all time points and regions, males consistently exhibited higher rates of both incidence and mortality than females. AYA = adolescents and young adults, GBD = Global Burden of Disease, UI = uncertainty interval.

We also compared the incidence and mortality rates of AYA GC across 21 GBD regions in 1990 and 2021, stratified by sex. In 1990, East Asia exhibited the highest age-standardized incidence rate (ASIR), particularly among males (up to 8.44 per 1,00,000), followed by Eastern Europe and Andean Latin America (Fig. 3A). Mortality patterns mirrored incidence, with High-income Asia Pacific, East Asia, and Eastern Europe showing the highest male mortality rates (Fig. 3B). In nearly all regions, males had significantly higher incidence and mortality than females.

By 2021, overall incidence and mortality rates had declined globally, but regional and gender disparities remained pronounced. High-income Asia Pacific continued to report the highest male incidence (4.55 per 1,00,000), while Andean Latin America and East Asia still exhibited elevated male mortality (Fig. 3C, D). Female rates also declined but at a slightly faster pace than males in most regions, widening the gender gap.

### 3.4. Impact of SDI on the burden of AYA gastric cancer

**Figure 4.**
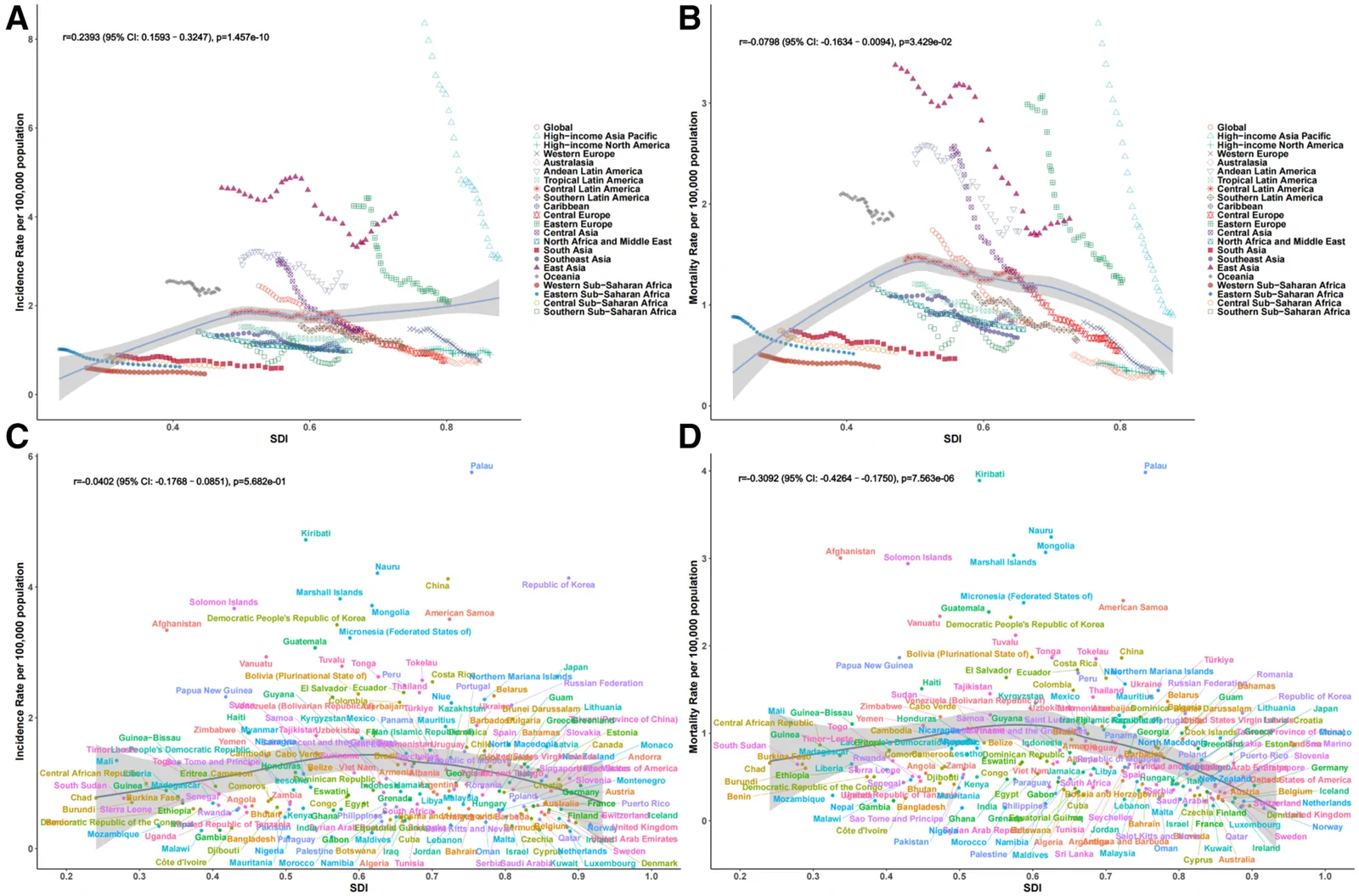
Association between SDI and AYA gastric cancer burden across global regions and countries, 1990 to 2021. This figure illustrates the relationship between the Socio-demographic Index (SDI) and the incidence and mortality rates of AYA gastric cancer. Panel A represents 21 GBD regional trends in incidence rate (per 1,00,000) and SDI from 1990 to 2021, showing a positive correlation (*r* = 0.2393, *P* < .001), especially in East Asia and high-income Asia Pacific regions. Panel B represents 21 GBD regional trends in mortality rate and SDI, revealing a weak negative correlation (r = −0.0798, *P* = .034), with considerable variability among regions. Panel C displays the association between incidence rate and SDI at the country level in 2021. No statistically significant correlation was observed (*r* = 0.0402, *P* = .56). Panel D displays the association between mortality rate and SDI in 2021, indicating a significant negative correlation (*r* = −0.3092, *P* < .001), with a higher mortality burden in low-SDI countries. AYA = adolescents and young adults, GBD = Global Burden of Disease, SDI = Socio-demographic Index.

We analyzed the association between the SDI and age-standardized incidence and mortality rates of AYA GC from both temporal and cross-sectional perspectives. Temporally, from 1990 to 2021, a positive correlation was observed between SDI and the incidence rate (*r* = 0.2393, 95% CI: 0.1593 to 0.3247, *P* < .001), indicating that more developed regions – particularly High-income Asia Pacific and East Asia – had persistently higher incidence burdens (Fig. 4A). In contrast, the association between SDI and mortality rate over time was slightly negative (*r* = −0.0798, 95% CI: −0.1634 to −0.0094, *P* = .034), suggesting modest improvements in mortality outcomes in higher-SDI settings (Fig. 4B).

Cross-sectionally, the 2021 data revealed no significant association between national SDI and incidence rates across 204 countries (*r* = 0.0402, 95% CI: −0.1768 to 0.0851, *P* = .562), as some low-SDI countries (e.g., Kiribati, Marshall Islands) reported unexpectedly high incidence, while many high-SDI nations (e.g., Republic of Korea, Japan) had relatively lower rates (Fig. 4C). However, a significant negative correlation was found between SDI and mortality rates (*r* = −0.3092, 95% CI: −0.4264 to −0.1750, *P* < .001), confirming that countries with lower socioeconomic development faced disproportionately higher death rates (Fig. 4D).

### 3.5. APC analysis for AYA gastric cancer

**Figure 5.**
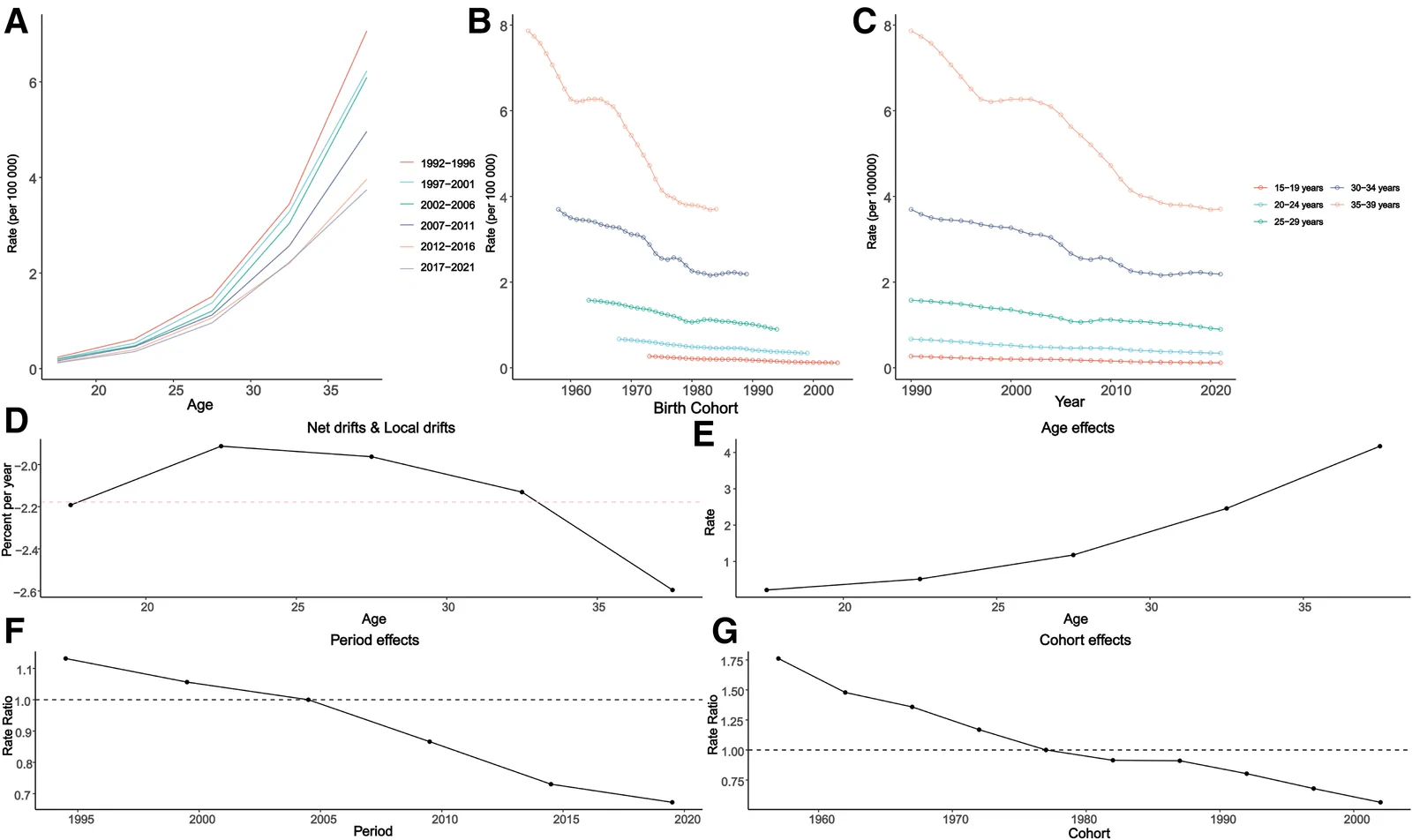
Age–period–cohort analysis of global AYA gastric cancer incidence rates, 1992 to 2021. This figure presents the age–period–cohort (APC) analysis of global incidence trends in AYA gastric cancer using 6 complete 5-year calendar periods from 1992–1996 to 2017–2021. Panel A: Age-specific incidence rates across different time intervals. Incidence consistently increased with age, peaking in the 35 to 39 age group. Panel B: Cohort-specific incidence trends stratified by birth year. The incidence rate was highest in individuals born in the 1960s and declined steadily in successive cohorts. Panel C: Period-specific incidence trends stratified by age group. All age groups showed declining incidence over time, with the most prominent decrease observed after 2010. Panel D: Net drift and local drift analysis. Net drift was estimated at −2.18% per year, indicating an overall annual decrease in incidence. Local drift values varied by age group. Panel E: Age effect showing a monotonic increase in incidence with increasing age. Panel F: Period effect with incidence rate ratio (RR) declining continuously from 1995 to 2020. Panel G: Cohort effect showing a peak RR among the 1960 birth cohort, followed by a steady decrease in more recent cohorts. APC = age–period–cohort, AYA = adolescents and young adults, RR = rate ratio.

The APC analysis revealed that the global incidence of AYA GC was significantly influenced by age, birth cohort, and time period effects (Fig. 5). Incidence rates increased sharply with age across all periods, peaking in the 35 to 39 age group (Fig. 5A). The highest incidence was observed during 1992 to 1996, with a progressive decline in subsequent periods. Cohort-specific trends showed that individuals born in the 1960s had the highest incidence rates, which steadily decreased across younger cohorts, with the 2000 cohort exhibiting the lowest rates (Fig. 5B). Period-specific trends across age groups demonstrated a gradual reduction in incidence after 2010 (Fig. 5C).

The net drift, reflecting the overall annual change in incidence, was estimated at −2.18% (Fig. 5D), while local drifts varied by age, with a steeper decline in older age groups. Age effects showed a positive correlation between age and incidence risk (Fig. 5E), whereas the period effect indicated a steady decline in incidence rate ratios from 1995 to 2020 (Fig. 5F). The cohort effect further confirmed a marked decrease in risk among individuals born after 1970, suggesting significant generational improvements in exposure and early detection (Fig. 5G).

### 3.6. Risk factors for AYA gastric cancer

**Figure 6.**
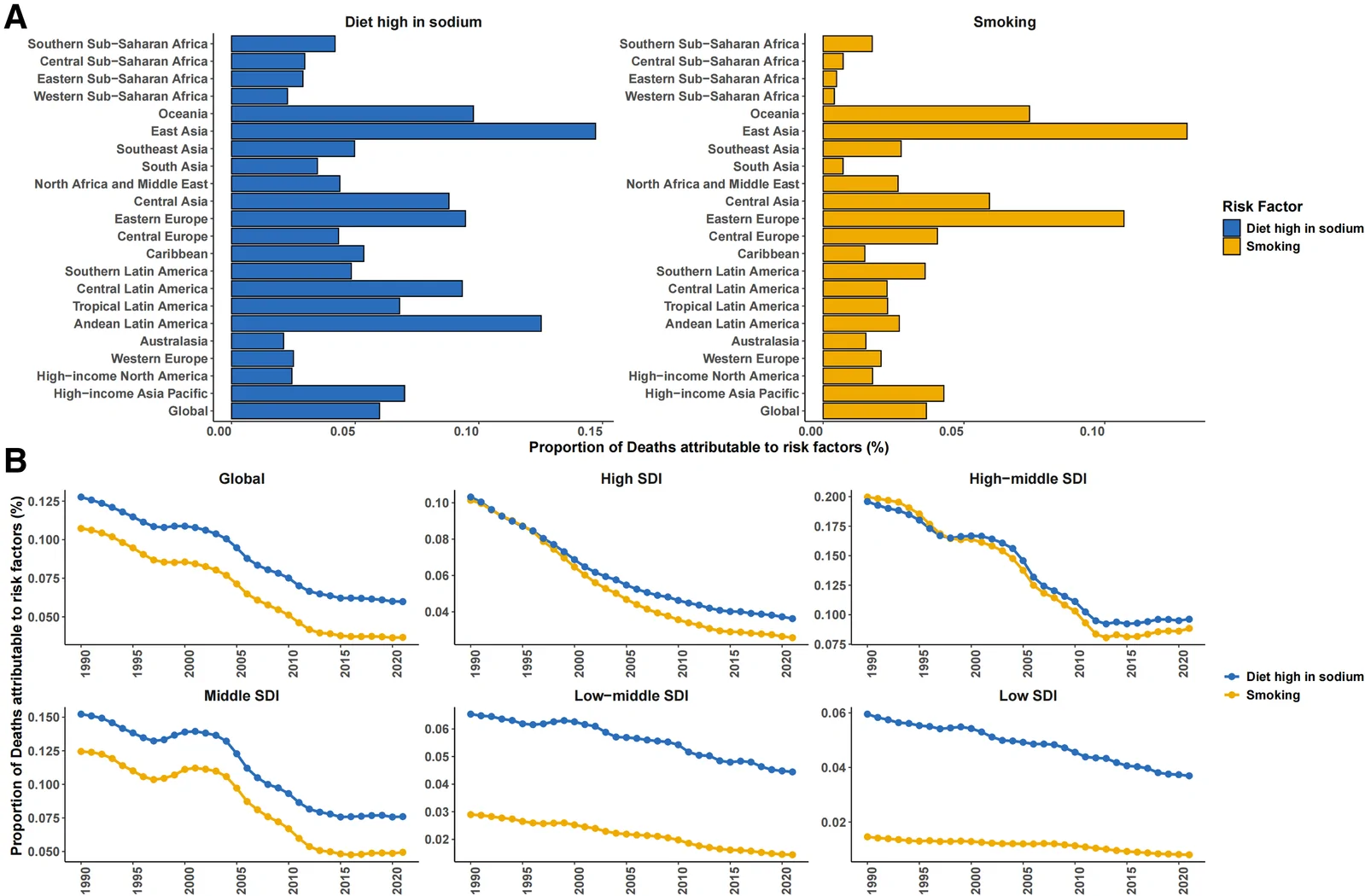
Trends and regional distribution of risk factors for AYA gastric cancer deaths, 1990 to 2021. This figure illustrates the GBD-attributed proportions of AYA gastric cancer deaths related to 2 selected risk factors, a diet high in sodium and smoking, based on the GBD 2021 Comparative Risk Assessment framework. Panel A: GBD-attributed proportions of deaths related to a diet high in sodium and smoking by GBD region in 2021. Panel B: Temporal trends in the GBD-attributed proportions of gastric cancer deaths related to a diet high in sodium and smoking from 1990 to 2021, stratified by SDI level. These estimates represent GBD-modeled attributable burdens and should not be interpreted as causal fractions newly estimated from individual-level data in the present study. AYA = adolescents and young adults, SDI = Socio-demographic Index.

In 2021, the GBD-attributed proportions of AYA GC deaths related to a diet high in sodium and smoking varied substantially across GBD regions (Fig. 6A). The GBD-attributed proportion related to a diet high in sodium was highest in East Asia, at approximately 15%, followed by Central Asia and Eastern Europe. The GBD-attributed proportion related to smoking was relatively higher in Central Asia and Eastern Europe, where it exceeded 10% in some regions.

Temporal analysis from 1990 to 2021 showed a global decline in the GBD-attributed proportions of AYA GC deaths related to both a diet high in sodium and smoking (Fig. 6B). High-SDI and high-middle-SDI regions experienced the most marked decreases, whereas reductions were less pronounced in low-SDI regions. These results describe regional and socioeconomic patterns in GBD-modeled attributable burden and should not be interpreted as causal effects estimated from individual-level data in the present study.

## 4. Discussion

Drawing on data from the GBD 2021 study, our analysis demonstrates a continued global decline in age-standardized incidence, mortality, and DALY rates of GC among AYAs (ages 15–39) from 1990 to 2021. However, this downward trend is accompanied by persistent disparities across sex, geographic regions, and sociodemographic strata. Application of an APC framework to the latest GBD release provides a detailed decomposition of temporal trends, while stratification by SDI highlights ecological patterns in the distribution of disease burden.

Disparities across SDI levels are pronounced. High-SDI regions, despite higher baseline incidence in 1990 (2.70 per 1,00,000), exhibited the steepest declines (EAPC: −2.57 for incidence, −3.55 for mortality, and −3.54 for DALYs). In contrast, low- and middle-SDI regions showed more modest improvements (e.g., incidence EAPC −1.09%). These ecological associations suggest that higher socioeconomic development correlates with faster burden reduction, potentially reflecting differences in healthcare access, risk factor exposure, and prevention efforts.^[22]^ However, as this is an aggregate-level analysis, such patterns are subject to ecological fallacy and do not permit causal inference at the individual level.^[23]^

Gender disparities remain evident, with males consistently experiencing a higher burden, although females showed slightly faster declines in some metrics. This may relate to differences in modifiable risk behaviors, such as a higher prevalence of high-salt diets, smoking, alcohol use, and *H pylori* infection among males in many settings. In contrast, adolescent females often adopt healthier behaviors earlier.^[24,25]^

APC analysis revealed a positive age effect peaking at 35 to 39 years, a net drift of −2.18% per year, and substantial risk reduction in cohorts born after 2000, which coincides with the global reduction in *H pylori* infection among children and adolescents.^[26]^ Consolidating low-risk profiles in emerging generations while addressing elevated age-related risks in older young adults could support continued progress.

From the perspective of the GBD comparative risk assessment framework, a diet high in sodium and smoking remained important GBD-attributed risk factors for AYA GC deaths, although their attributed proportions declined over time, particularly in high-SDI regions. In 2021, the GBD-attributed proportion of AYA GC deaths related to a diet high in sodium was approximately 15% in East Asia, while the GBD-attributed proportion related to smoking exceeded 10% in parts of Central and Eastern Europe.^[27-29]^ Modeling studies suggest that accelerating anti-smoking measures could avert an additional 2 billion life years lost by 2050.^[30]^ Collectively, these trends highlight the success of salt reduction and tobacco control efforts in high-development settings, while the persistent burden in lower-SDI areas underscores the urgent need for targeted prevention strategies. However, because the present study used aggregate GBD estimates, these findings should be interpreted as population-level attributable burden estimates within the GBD framework, rather than causal effects directly estimated from individual-level data.

A key strength of this study is the use of the GBD 2021 dataset, enabling standardized comparisons across 204 countries over 3 decades and a comprehensive assessment of incidence, mortality, and DALYs in the AYA population. The combination of EAPC and APC modeling provides complementary views of temporal change. Several limitations should be emphasized. First, GBD estimates rely on heterogeneous data inputs and modeling assumptions; uncertainty may be greater in locations with limited primary data. Second, this is a descriptive, ecological analysis based on aggregate GBD estimates; therefore, associations with SDI and GBD-attributed risk factors should not be interpreted as individual-level causal relationships. The risk factor estimates for a diet high in sodium and smoking were derived from the GBD comparative risk assessment framework and represent modeled attributable burdens rather than causal effects estimated in the present study. Third, the GBD framework does not fully capture clinically relevant heterogeneity (e.g., cardia vs non-cardia disease, histologic and molecular subtypes), and risk factor attribution is constrained by the granularity of available exposure categories.^[31-33]^

These findings emphasize the importance of sustaining prevention in low-risk younger cohorts and intensifying efforts in high-burden regions and demographic groups. Future research should prioritize prospective studies to explore individual-level mechanisms and evaluate targeted interventions. Establishment of high-quality registries integrating relevant data could further inform strategies to reduce persistent disparities.

## 5. Conclusion

In summary, the global burden of GC among AYAs declined from 1990 to 2021, yet substantial regional and demographic disparities persist. These findings highlight the need for context-specific surveillance and resource allocation, while causal mechanisms require confirmation in analytic epidemiologic studies.

## Acknowledgments

We appreciate the high-quality data provided by the Global Burden of Disease Study 2021 collaborators. We also would like to thank the editors and reviewers for their time and effort dedicated to improving the report of this work.

## Author contributions

**Conceptualization:** Zhen-Zhen Peng.

**Data curation:** Zhen-Zhen Peng.s

**Formal analysis:** Zhen-Zhen Peng.

**Funding acquisition:** Tao Zhang.

**Investigation:** Maoqiang Liu.

**Methodology:** Maoqiang Liu, Wei Liu.

**Project administration:** Wei Liu, Tao Zhang.

**Software:** Maoqiang Liu.

**Supervision:** Tao Zhang.

**Validation:** Maoqiang Liu, Wei Liu.

**Writing – original draft:** Maoqiang Liu, Zhen-Zhen Peng, Wei Liu, Tao Zhang.

**Writing – review & editing:** Maoqiang Liu, Zhen-Zhen Peng, Wei Liu, Tao Zhang.
